# Efficacy of Vitamin E Supplementation During Pregnancy on the Vitamin E Nutritional Status of the Mother–Child Dyad: A Systematic Review and Meta‐Analysis of Randomized Controlled Trial

**DOI:** 10.1002/fsn3.71677

**Published:** 2026-03-22

**Authors:** Byanca Rodrigues Carneiro, Anny Cristine de Araújo, Yvi Melo Sisinano, Amanda Freitas de Oliveira, Adriana Augusto de Rezende, Karla Danielly da Silva Ribeiro

**Affiliations:** ^1^ Postgraduate Program in Applied Sciences to Women's Health Federal University of Rio Grande Do Norte Natal Rio Grande do Norte Brazil; ^2^ Postgraduate Program in Health Sciences Federal University of Rio Grande Do Norte Natal Rio Grande do Norte Brazil; ^3^ Department of Nutrition Federal University of Rio Grande Do Norte Natal Rio Grande do Norte Brazil; ^4^ Postgraduate Program in Nutrition Federal University of Rio Grande Do Norte Natal Rio Grande do Norte Brazil; ^5^ Department of Clinical and Toxicological Analyses Federal University of Rio Grande Do Norte Natal Rio Grande do Norte Brazil

**Keywords:** alpha‐tocopherol, clinical trials, maternal health, maternal‐child dyad, meta‐analysis, pregnancy, supplementation

## Abstract

The objective of this systematic review and meta‐analysis is to investigate the effects of vitamin E supplementation on maternal and neonatal vitamin E status. Studies were systematically searched in PubMed, Scopus, Web of Science, EMBASE, LILACS, and the Cochrane Library. Randomized controlled trials (RCTs) comparing vitamin E supplementation in pregnant women with placebo were included. Study selection, data extraction, and risk of bias assessment using the Cochrane Risk of Bias tool were independently performed by reviewers. Meta‐analyses were conducted using RevMan 5.4, and the certainty of evidence was assessed using the GRADE approach. Six RCTs including 3.823 pregnant women met the inclusion criteria. Vitamin E supplementation significantly increased maternal serum α‐TOH concentrations (SMD = 4.89; 95% CI: 2.89–6.89; *p* < 0.001), although substantial heterogeneity was observed (*I* ^2^ = 99%). Maternal serum levels of vitamin E were measured at baseline and postpartum. No significant effects were found for gestational age at birth (SMD = 0.04 weeks; 95% CI: −0.03 to 0.11; *I* ^2^ = 0%; *p* = 0.29) or birth weight (SMD = −0.02 g; 95% CI: −0.09 to 0.04; *I* ^2^ = 62%; *p* = 0.49). Overall, the studies showed a low risk of bias, with evidence certainty ranging from low to moderate. Meta‐analysis results indicate that vitamin E supplementation during pregnancy increases maternal α‐TOH levels but shows no consistent clinical benefits for neonatal outcomes. Evidence is limited and heterogeneous, highlighting the need for well‐designed trials to clarify its role.

## Graphical abstract

Summary of the systematic review and meta‐analysis evaluating the efficacy of vitamin E supplementation during pregnancy on the vitamin E nutritional status of the mother–child dyad.

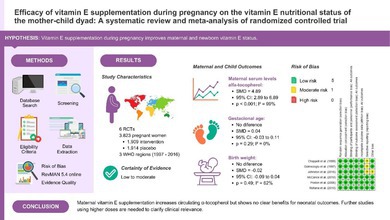

## 1 Introduction

Vitamin E is a group of fat‐soluble compounds that includes tocopherols and tocotrienols, each subdivided into four distinct forms (α‐, β‐, γ‐, and δ‐). Among these, alpha‐tocopherol (α‐TOH) stands out as the most biologically active form and is essential for humans due to its potent antioxidant effect. This compound plays a crucial role in protecting cell membranes against oxidation and lipid peroxidation, preventing cellular damage (Galli et al. 2017; Lee and Han 2018; Traber and Head 2021).

During pregnancy, vitamin E is essential for protecting cells from oxidative damage, supporting maternal immunity, and preventing infections (Traber and Stevens 2011). It also plays a key role in embryogenesis, metabolism, and gene regulation, with α‐TOH specifically preventing lipid peroxidation and supporting early fetal development (Traber 2024). Nutritional demands increase during this period, but populations with limited access to vitamin E–rich foods, such as oils, nuts, and seeds, are at risk of deficiency (Rebouças et al. 2019; Busso et al. 2021).

Recent studies suggest that low vitamin E levels, defined as serum concentrations below 12 μmol/L (Péter et al. 2015), increase maternal oxidative stress, thereby raising the risk of complications such as preeclampsia and placental abruption (Debier and Larondelle 2005). In fetuses, deficiency is linked to higher susceptibility to infections, IUGR, and neurological damage, especially in preterm infants. These findings underscore the need to investigate the effects of circulating α‐TOH and vitamin E supplementation on pregnancy outcomes (Debier and Larondelle 2005; Traber 2014, 2021). A systematic review by Rumbold et al. found that vitamin E combined with other supplements did not prevent fetal or neonatal death, preterm birth, preeclampsia, or IUGR, but the lack of data on isolated α‐TOH prevents conclusions about its independent effect (Rumbold et al. 2015).

A study by Ribeiro et al. (2016). showed that 19% of pregnant women had low levels of α‐TOH, while 90% of newborns had reduced concentrations of this vitamin in the umbilical cord. The research highlighted that newborns of mothers with insufficient α‐TOH levels (< 16.2 mmol/L) had significantly lower vitamin E levels in the umbilical cord, demonstrating the correlation between maternal and neonatal levels. A systematic review by Wang et al. found that low maternal vitamin E was associated with higher risks of preeclampsia, IUGR, preterm birth, low birth weight, and prematurity‐related complications (Wang et al. 2023).

Although vitamin E supplementation may benefit maternal and neonatal health, its isolated use is poorly studied. Deficiency is linked to adverse outcomes, and it remains unclear whether α‐TOH supplementation effectively improves the mother–child dyad's nutritional status (Rumbold et al. 2015). This systematic review and meta‐analysis aimed to evaluate the effectiveness of vitamin E supplementation during pregnancy on the nutritional status of the mother–child dyad.

## 2 Methods

### 2.1 Protocol Registration

We conducted the study following the Preferred Reporting Items for Systematic Reviews and Meta‐Analysis (PRISMA) guidelines (Table S1) (Page et al. 2021). The study protocol was prospectively registered on the International Prospective Register of Systematic Reviews (PROSPERO) under number CRD42021250849.

### 2.2 Eligibility Criteria

The eligibility criteria were based on the PICOS strategy (population, intervention, comparison, outcomes, and study design). The population (P) comprised pregnant women. The intervention (I) included supplementation with vitamin E, either alone or in combination with up to two additional micronutrients. The comparison (C) consisted of placebo administration. The outcomes (O) referred to maternal or neonatal vitamin E status, assessed through serum or tissue levels. Regarding the study design (S), only randomized controlled trials (RCTs) were included. Studies were excluded if they were conducted in animals, if the intervention lasted less than one week, if they did not provide data on vitamin E, or if they were observational studies, reviews, conference abstracts, duplicates, or lacked full‐text availability.

### 2.3 Search Strategy

The search strategy was developed using a combination of Medical Subject Headings (MeSH), keywords, and Boolean operators to maximize retrieval. It was adapted for each database according to the specific guidelines of each platform. The following databases were searched: PubMed, Scopus, Web of Science, EMBASE, LILACS, and Cochrane Li‐brary. Filters were applied, when available, to restrict results to human studies. The initial search was conducted in March 2024 and updated in November 2024. The complete search strategy is presented in Table S2.

### 2.4 Selection of Studies

Study selection was conducted using Rayyan software (https://www.rayyan.ai/) (Ouzzani et al. 2016), where duplicates were initially removed. Titles and abstracts were independently screened by four investigators (B.R.C., A.C.A., Y.M.S., and A.F.O.), with a fifth investi‐gator (K.D.S.R.) consulted in cases of disagreement. Full texts of potentially eligible arti‐cles were then reviewed in a similar process. For studies in which the full text was una‐vailable, corresponding authors were contacted up to three times. In addition, the refer‐ence lists of the included articles were manually screened to identify articles potentially missed in the search. The search and selection process was presented in a PRISMA flow diagram (Page et al. 2021).

### 2.5 Data Extraction

Following full‐text review, data were manually extracted by two independent re‐viewers (B.R.C. and A.C.A.) and verified by a third reviewer (K.D.S.R.). The information was organized in a spreadsheet and included: first author, year of publication, country, study design, number of participants, mean age, gestational age at the beginning of the intervention, maternal and neonatal comorbidities, maternal supplementation (type, form/route, composition, dose, timing, duration, tolerance, and safety), serum vitamin E levels, vitamin E biomarkers, statistical analysis, and main outcomes.

In data analysis, categorical variables were represented as counts and percentages, and continuous variables were reported as mean and standard deviation (SD). If needed, the median and interquartile range (IQR) were converted to mean and SD. For the results presented in graphical format, the WebPlotDigitiser software online (https://automeris.io/) was used to extract the values. (Drevon et al. 2017) When additional information was required, the corresponding author was contacted by email up to three times. Variables that were investigated but not reported were recorded as “No information,” while variables not assessed in the studies were classified as “Not as‐sessed.”

### 2.6 Data Analysis and Synthesis

The meta‐analysis was performed when at least three studies reported data for a given outcome. The data synthesis was performed independently by two reviewers (B.R.C. and A.C.A.), and all analyses were conducted using the statistical package in the desktop version of the Cochrane Review Manager (RevMan), version 5.4 (2020). Associations were ana‐lyzed with fixed and random‐effects models. Continuous data were pooled as standard‐ized mean difference (SMD) with a 95% confidence interval (CI). In contrast, dichotomous data were pooled as risk ratio (RR) with a 95% CI. Significance was determined by the Chi‐Square tests (*χ* ^2^) or *z* test effect (*p* < 0.05), and heterogeneity by the *I* ^2^ statistic (0%–100%). (Deeks et al. 2019) Results were presented in forest plots. Studies not suitable for meta‐analysis were qualita‐tively analyzed in the systematic review.

### 2.7 Quality of Evidence

The GRADE system (Grading Recommendations, Assessment, Development, and Evaluation) was used to assess the quality of the results. Studies were classified as low, moderate, or high quality. Two independent reviewers (B.R.C. and A.C.A.) conducted the assessment using the online GRADEpro software (https://www.gradepro.org/), with disa‐greements resolved through discussion with a third reviewer (K.D.S.R.). Decisions to downgrade the certainty of evidence followed GRADE guidelines. (Zeng et al. 2022; Guyatt, Oxman, Vist, et al. 2011; Guyatt et al. 2023; Guyatt, Oxman, Kunz, et al. 2011) A summary table was created, showing the judgment for each factor affecting evidence certainty and the final rating for each outcome.

### 2.8 Risk of Bias Assessment

A summary table was created, showing the judgment for each factor affecting evidence certainty and the final rating for each outcome (Sterne et al. 2019), which contains six domains: (1) random sequence generation (2), deviation from the in‐tended interventions (3), missing outcome data (4), measuring outcome (5), selection of the reported outcome, and (6) other bias. The investigators' conclusions are classified as “low risk,” “no information,” “some concerns,” or “high risk” of bias for each of these topics. A third investigator (K.D.S.R.) reanalyzed the disagreements and resolved them. The RoB 2.0 tool summary and graph were produced using the RevMan version 5.4.

## 3 Resuls

### 3.1 Study Selection

**FIGURE 1:**
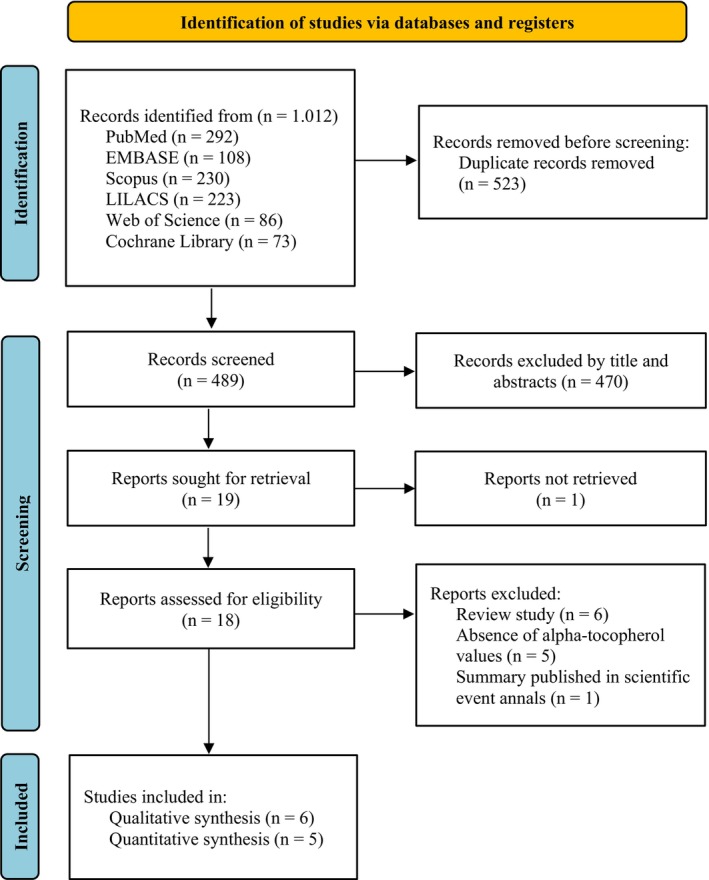
PRISMA flowchart of the study selection process.

The search identified 1.012 potentially relevant publications. After removing 523 duplicates, 489 records remained for title and abstract screening. Of these, 19 articles were assessed in full text. One article was unavailable, and no response was obtained from the authors (Laskowska‐Klita et al. 2004). Among the 18 articles reviewed, 12 did not meet the eligibility criteria (Table S3), resulting in the inclusion of six RCTs (Gülmezoǧlu et al. 1997; Chappell et al. 1999; Poston et al. 2006; Rohana et al. 2010; McCance et al. 2010; Johnston et al. 2016). No additional studies were retrieved from reference lists. These details are shown in Figure 1.

### 3.2 Study Characteristics

A summary of the characteristics and main results of each study is presented in Table 1. he articles were published between 1997 and 2016 and included participants from the United Kingdom (*n* = 4; 66.7%) (Chappell et al. 1999; Poston et al. 2006; McCance et al. 2010; Johnston et al. 2016), Malaysia (*n* = 1; 16.7%) (Rohana et al. 2010) and South Africa (*n* = 1; 16.7%). (Gülmezoǧlu et al. 1997) Among the six included articles, a total of 1.909 pregnant women received the intervention, and 1.914 pregnant women received the placebo group. Participants had clinical conditions such as preeclampsia (Gülmezoǧlu et al. 1997; Chappell et al. 1999; McCance et al. 2010; Johnston et al. 2016), diabetes mellitus (Chappell et al. 1999; Poston et al. 2006; McCance et al. 2010; Johnston et al. 2016) and antiphospholipid syndrome. (Chappell et al. 1999; Poston et al. 2006) One study did not specify the participants clinical conditions. (Rohana et al. 2010) Only one study reported participants education level (Poston et al. 2006), provided information on socioeconomic status, dietary habits, or vitamin E intake.

**TABLE 1:** Characteristics of included studies in the review (*n* = 6).

Author (year); country	Participants, No.	Mean age (years)	Clinical condition	Gestational age at intervention start (weeks) Mean ± SD	Intervention			Comparator	Safety indicators
					Duration of intervention^c^	Vitamin (s)	Daily dose		
Gülmezoǧlu et al. (1997); South Africa	Intervention (*n* = 27) Control (*n* = 29)	29	Severe PE	30 (25–32)^b^	2–6 weeks (14–42 days)	Vitamin E Vitamin C Allopurinol	800 IU/day 1000 mg/day 200 mg/day	NI	Acne, transient weakness, and skin rash after childbirth
Chappell et al. (1999); United Kingdom	Intervention (*n* = 141) Control (*n* = 142)	29	Risk for PE, HAS, DM, lupus/SAF	17 to 23^a^	17–23 weeks (119–161 days)	RRR‐α‐TOH Vitamin C	400 IU/day 1000 mg/day	Soybean oil	NI
Poston et al. (2006); United Kingdom	Intervention (*n* = 1199) Control (*n* = 1205)	31	Previous PE, HELLP syndrome or eclampsia, DM, SAF and CKD	18.6 ± 2.5	16–23 weeks (112–161 days)	RRR‐α‐TOH Vitamin C	400 IU/day 1000 mg/day	Sunflower oil	Clinical emergencies
Rohana et al. (2010); Malaysia	Intervention (*n* = 136) Control (*n* = 126)	SI	SI	12 to 16^a^	22–26 weeks (154–182 days)	Tocotrienol	10 mg	Superolein	NI
McCance et al. (2010); United Kingdom	Intervention (*n* = 379) Control (*n* = 382)	30	DM, previous PE and hypertension	14.3 ± 3.6	15–29 weeks (105–203 days)	α‐TOH Vitamin C	400 IU/day 1000 mg/day	Olive oil	NI
Johnston et al. (2016); United Kingdom	Intervention (*n* = 27) Control (*n* = 30)	31	PE and DM	14.3 ± 3.6	15–29 weeks (105–203 days)	α‐TOH Vitamin C	400 IU/day 1000 mg/day	Olive oil	NI

*Note:* The intervention duration interval was calculated by subtracting the mean gestational age at delivery from the intervention strar period.

Abbreviations: CKD, chronic kidney disease; DM, diabetes mellitus; HAS, systemic arterial hypertension; HELLP, hemolysis, elevated liver enzymes and low platelets; IU, international units; NI, not informed; PE, preeclampsia; RRR‐α‐TOH, RRR‐alpha‐tocopherol; SAF, antiphospholipid syndrome; SD, standard deviation; α‐TOH, alpha‐tocopherol.

^a^ Intervention start interval.

^b^ Value expressed as median [range].

^c^ In all included RCTs, the intervention was carried out until delivery.

### 3.3 Characteristics of Vitamin E Supplementation

The intervention period in the studies ranged from the 8th to the 32nd week of gestation and continued until delivery. Five RCTs (Gülmezoǧlu et al. 1997; Chappell et al. 1999; Poston et al. 2006; Rohana et al. 2010; McCance et al. 2010) (83.3%) combined vitamin E with vitamin C, while one study (16.7%) used allopurinol (Gülmezoǧlu et al. 1997). Most studies administered 400 IU/day of vitamin E, with one trial (33.3%) using 800 IU/day (Gülmezoǧlu et al. 1997) and another using 10 mg/day of tocotrienol (Rohana et al. 2010). Only study (Chappell et al. 1999) justified the 400 IU α‐TOH dose, citing its effectiveness in reducing LDL oxidation and benefits in patients with coronary artery disease. All RCTs were placebo‐controlled. Comparison vehicles included soybean oil (Chappell et al. 1999), and sunflower oil (Poston et al. 2006), superolein (Rohana et al. 2010), olive oil (McCance et al. 2010; Johnston et al. 2016), though no study provided detailed justification for their choice.

### 3.4 Biomarkers of Vitamin E and Supplementation Adherence

Among the biomarkers analyzed in the RCTs, most focused on serum α‐TOH (Chappell et al. 1999; Poston et al. 2006; McCance et al. 2010; Johnston et al. 2016), with three studies adjusting this biomarker for cholesterol levels (reported as μmol/mmol) (Poston et al. 2006; McCance et al. 2010; Johnston et al. 2016). Two studies did not specify the biomarker analyzed (Gülmezoǧlu et al. 1997; Rohana et al. 2010). Serum α‐TOH was used due to its antioxidant properties, which can reduce oxidative stress linked to pregnancy complications, such as preeclampsia (Gülmezoǧlu et al. 1997; Chappell et al. 1999; McCance et al. 2010; Johnston et al. 2016), neonatal jaundice (Rohana et al. 2010), and diabetes mellitus (Chappell et al. 1999; Poston et al. 2006; McCance et al. 2010; Johnston et al. 2016).

Vitamin E was measured in different biological samples: maternal blood (reported as μg/mL or μmol/L) (Gülmezoǧlu et al. 1997; Chappell et al. 1999; Poston et al. 2006; McCance et al. 2010; Johnston et al. 2016), umbilical cord (μmol/L) (Rohana et al. 2010; Johnston et al. 2016), and central and peripheral placenta tisue (μmol/L) (Johnston et al. 2016). Maternal blood was primarily used to directly assess antioxidant levels in the mother, whereas umbilical cord and placental samples were analyzed to evaluate antioxidant enzymes and lipid peroxidation, reflecting oxidative status in placental tissue (Johnston et al. 2016).

In five trials (83.3%) (Gülmezoǧlu et al. 1997; Chappell et al. 1999; Poston et al. 2006; Rohana et al. 2010; McCance et al. 2010), serum vitamin E concentrations were quantified using High‐Performance Liquid Chromatography (HPLC), a method known for its precision and sensitivity in measuring fat‐soluble compounds. One study did not specify the method used (Johnston et al. 2016). It is worth noting that the included studies did not provide detailed information on adherence to supplementation.

### 3.5 Effect of Supplementation on the Mother–Child Dyad

Table 2 summarizes the characteristics of vitamin E supplementation in pregnant women and its effects on maternal and neonatal serum levels. Overall, supplementation led to increased maternal α‐TOH concentrations compared to placebo, with reported rises ranging from 5% to 30% (Poston et al. 2006; Johnston et al. 2016) and reaching up to 100% with higher doses (800 IU/day) (Gülmezoǧlu et al. 1997). These results suggest a dose‐dependent effect on maternal vitamin E status. Poston et al. (2006). observed consistently higher α‐TOH levels throughout pregnancy in supplemented women, including those who later developed preeclampsia. Adverse effects were generally mild; Gülmezoǧlu et al. (1997). reported acne, transient weakness, and postpartum rash. Poston et al. (2006). also noted a higher number of unexplained fetal deaths (≥ 24 weeks) in the intervention group; however, these occurred primarily among very preterm and/or low birth weight infants and were not attributed to the supplementation. Rates of congenital anomalies were similar between groups, and neonatal mortality was 4% in the intervention group versus 3% in the control group.

**TABLE 2:** Nutritional status of vitamin E in supplemented pregnant women and their newborns (*n* = 6).

Author, (year); country	Biomarker; biological sample	Pregnant women				Newborns		Main outcomes on vitamin E nutritional status	Other findings related to vitamin E supplementation
		Baseline serum levels^e^ of α‐TOH		Serum levels^e^ of α‐TOH after supplementation		Baseline serum levels^e^ of α‐TOH			
		Intervention	Comparator	Intervention	Comparator	Intervention	Comparator		
Gülmezoǧlu et al. (1997); South Africa^a^	NI; Sangue	23.6 μmol/L (9–42.4)^a^	22.8 μmol/L (11.8–36.6)^a^	38.8 μmol/L (5.8–83.5)^a^	27.0 μmol/L (14–50.5)^a^	N/A	N/A	The intervention group had higher α‐TOH levels during pregnancy and postpartum compared to the control group (*p* = 0.003)	The intervention group had lower uric acid levels, but perinatal outcomes (Apgar scores, preterm birth, birth weight, ICU admission) did not differ between groups
Chappell et al. (1999); United Kingdom	α‐TOH; Sangue	26 μmol/L	30 μmol/L	50 μmol/L	34 μmol/L	N/A	N/A	The intervention group had a 41% increase in α‐TOH at the end of pregnancy. Preeclampsia occurred in 8% vs. 17% (placebo), with a 61% lower risk in the supplemented group (OR = 0.39, *p* = 0.02)	There were no significant differences between the groups regarding the number of SGA newborns (*p* = 0.12). There were no significant differences in perinatal characteristics between the vitamin and placebo groups
Poston et al. (2006); United Kingdom	α‐TOH/colesterol; Sangue	5 to 7 μmol/mmol^b^	6 to 7 μmol/mmol^b^	9 to 10 μmol/mmol^b^	6 to 7 μmol/mmol^b^	N/A	N/A	The intervention group showed higher α‐TOH concentrations throughout pregnancy, including those with preeclampsia, compared to the placebo group	The intervention group had more cases of LBW, SGA, hospital admission, MV, bronchopulmonary dysplasia, neonatal mortality, and congenital malformations, but evidence was insufficient to confirm a significant effect of supplementation
Rohana et al. (2010); Malaysia	Vitamina E; Cordão umbilical	N/A	N/A	N/A	N/A	1.78 μg/mL^c^	1.70 μg/mL^c^	The vitamin E levels in the umbilical cord blood did not differ significantly between the groups (*p* = 0.81)	No significant differences were observed between groups in sex distribution, gestational age at delivery, maternal blood type, cause of jaundice, hospitalization rate or duration, or phototherapy
McCance et al. (2010); United Kingdom^d^	α‐TOH/colesterol; Sangue	6.15 (1.24) μmol/mmol	6.19 (1.20) μmol/mmol	8 to 9 μmol/mmol	5 to 6 μmol/mmol	N/A	N/A	The intervention group showed higher concentrations of α‐TOH/cholesterol during pregnancy (*p* < 0.0001)	Supplementation did not reduce the risk of preeclampsia, gestational hypertension, or LBW, and no significant adverse effects were observed. Infant outcomes were similar, although preterm births were fewer in the intervention group
Johnston et al. (2016); United Kingdom	α‐TOH/colesterol; Sangue	6.1 (1.2) μmol/mmol	6.2 (1.4) μmol/mmol	7.3 (2.3) μmol/mmol	5.8 (1.3) μmol/mmol	N/A	N/A	The intervention group showed higher α‐TOH/cholesterol concentrations in the third trimester (*p* = 0.01), but no significant effect on α‐TOH levels in umbilical cord blood and the placenta	Supplementation did not increase vitamin E or C in the umbilical cord or placenta, nor alter antioxidant enzymes or lipid peroxidation, but oxidative stress was higher in preeclamptic placentas

Abbreviations: ICU, intensive care unit; LBW, low birth weight; MV, mechanical ventilation; N/A, not assessed/presented; NI, no information; SGA, small for gestational age; α‐TOH, alpha‐tocopherol; γ‐TOH, gamma‐tocopherol.

^a^ Values in median (95% CI).

^b^ Estimated values presented in the figure of the article.

^c^ The study does not specify which form of vitamin was analyzed in the umbilical cord. Due to the lack of this information, it was not possible to convert the data to mmol.

^d^ Only study that did not describe the analytical method.

^e^ The determination of vitamin E in the studies was performed using the HPLC method (high‐performance liquid chromatography).

No significant differences were observed in α‐TOH levels in umbilical cord blood or placental tissue (Rohana et al. 2010; Johnston et al. 2016). Despite higher maternal α‐TOH levels, neonatal clinical outcomes did not differ (McCance et al. 2010). In women with preeclampsia, supplementation was linked to increased neonatal complications, including low birth weight and hospitalizations (Poston et al. 2006).

### 3.6 Meta‐Analysis

**FIGURE 2:**
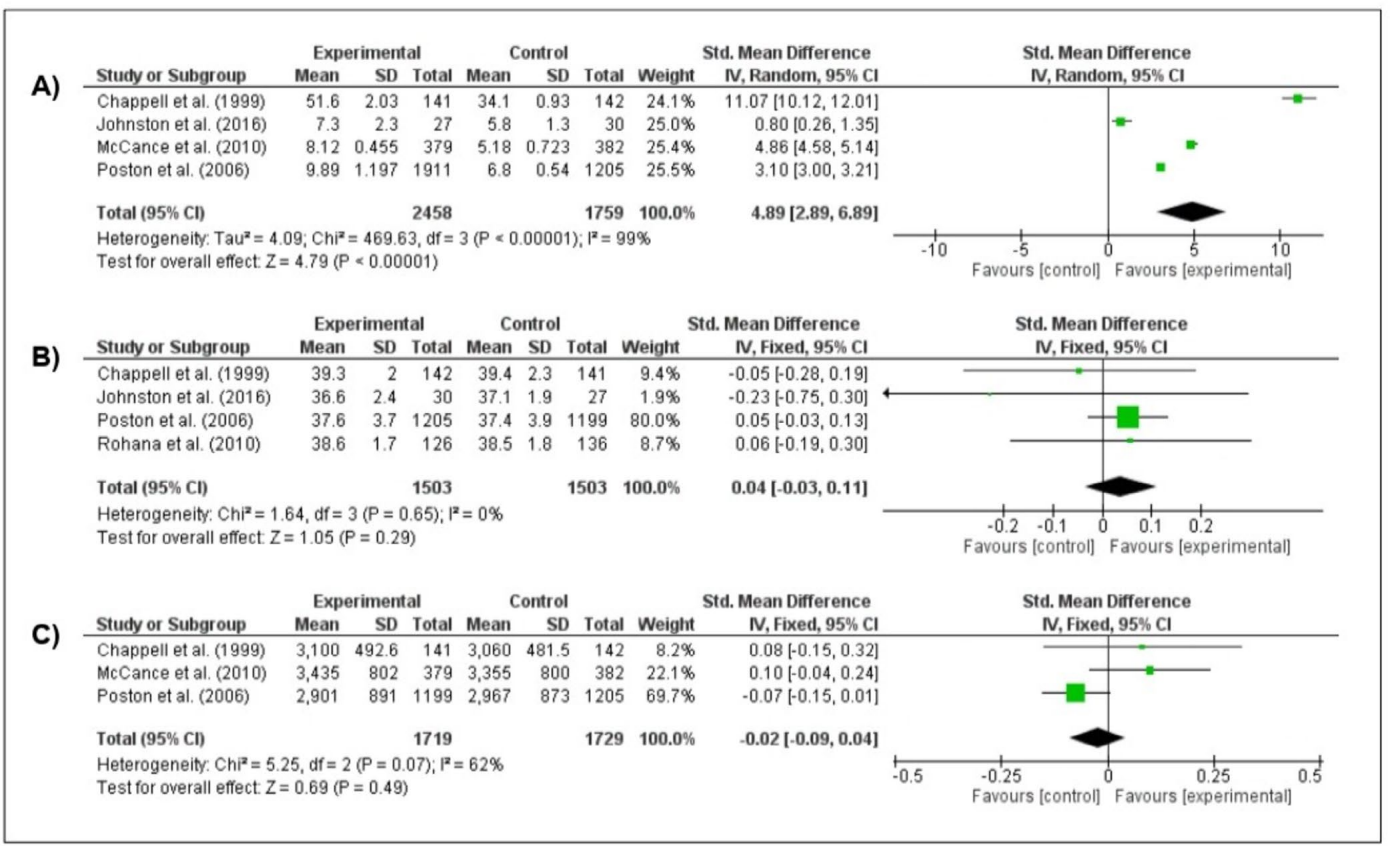
Forest plots showing the effects of supplementation on mother–child dyad outcomes. (A) Serum α‐tocopherol levels, (B) gestational age at birth, and (C) birth weight.

The meta‐analysis of four studies (Chappell et al. 1999; Poston et al. 2006; McCance et al. 2010; Johnston et al. 2016) evaluated the effects of vitamin E supplementation on maternal serum α‐TOH levels. High heterogeneity was observed among the studies (*I* ^2^ = 99%, *p* < 0.001); therefore, a random‐effects model was applied. All studies reported increases in maternal α‐TOH following supplementation, and the meta‐analysis demonstrated a significant effect of the intervention (SMD = 4.89; 95% CI: 2.89–6.89, *p* < 0.001) (Figure 2A).

In contrast, data from four studies (Chappell et al. 1999; Poston et al. 2006; Rohana et al. 2010; Johnston et al. 2016) assessing the effect of vitamin E supplementation on gestational age at birth showed no significant association (SMD = 0.04 weeks; 95% CI: −0.03 to 0.10; *I* ^2^ = 0%; *p* = 0.29) (Figure 2B). Similarly, three studies (Chappell et al. 1999; Poston et al. 2006; McCance et al. 2010) evaluating birth weight found no significant effect of supplementation (SMD = −0.02 g; 95% CI: −0.09 to 0.04; *I* ^2^ = 62%; *p* = 0.49) (Figure 2C). These results indicate that vitamin E supplementation did not significantly impact gestational duration or neonatal birth weight.

### 3.7 Certainty of Evidence

The certainty of evidence assessed by the GRADE approach was rated as moderate for maternal serum α‐TOH levels and gestational age at birth, and low for birth weight (Table 3). The lower certainty was mainly influenced by penalties in domain five of the risk of bias tool, as some studies evaluated only secondary outcomes and presented methodological limitations that reduced confidence in the findings.

**TABLE 3:** Grade certainty of the evidence.

Outcomes	Number of participants (studies) Follow‐up	Certainty of the evidence (GRADE)	Relative effect (95% CI)
Serum α‐TOH level	4.273 (5 RCTs)	⨁⨁⨁◯ Moderate^a^, ^b^	4.89 (2.89–6.89)
Gestational age at birth	3.006 (4 RCTs)	⨁⨁⨁◯ Moderate^c^	0.04 (−0.03–0.11)
Birth weight	3.448 (3 RCTs)	⨁⨁◯◯ Low^d^	−0.02 (−0.09–0.04)

Abbreviations: CI, confidence interval; GRADE, grading of recommendations, Assessment, Development, and Evaluation.

^a^ The value of *I* ^2^ was high.

^b^ The funnel plot indicates publication bias.

^c^ Penalty in domain five of the risk of bias assessment.

^d^ The outcomes evaluated are secondary.

### 3.8 Risk of Bias

**FIGURE 3:**
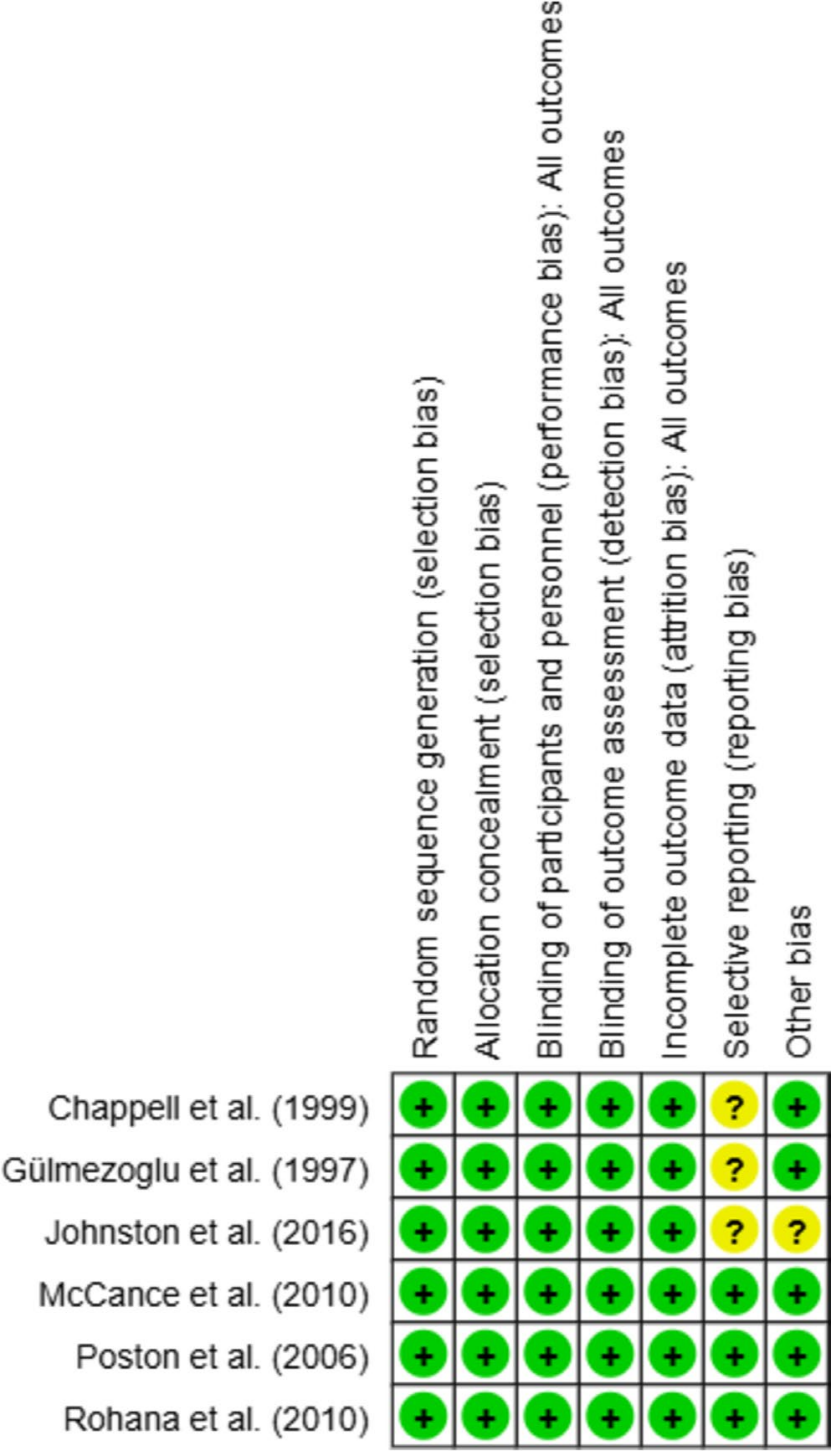
Risk of bias assessments for randomized controlled trials included.

Among the six included RCTs, three studies (Chappell et al. 1999; Poston et al. 2006; Rohana et al. 2010) were rated as having a low risk of bias across all assessed domains, reflecting a robust methodological approach. However, three trials (Gülmezoǧlu et al. 1997; Chappell et al. 1999; Johnston et al. 2016) deviated from pre‐established protocols, resulting in a higher risk of bias. The summary plot for the domains shows that deviations from intended interventions and incomplete outcome data contributed most to concerns regarding bias (Figure 3).

## 4 Discussion

This systematic review highlights the limited and methodologically heterogeneous evidence regarding the effects of vitamin E supplementation during pregnancy on maternal α‐TOH levels and neonatal outcomes. To our knowledge, this is the first review specifically designed to assess the effectiveness of supplementation on maternal circulating α‐TOH concentrations within the maternal–infant context, addressing a gap left by previous reviews focused on broader antioxidant strategies.

Overall, the synthesis of six RCTs demonstrated that vitamin E supplementation significantly increases maternal serum α‐TOH levels. However, despite this clear biochemical effect, the clinical relevance of supplementation remains uncertain. Methodological limitations were common across studies, including the absence of rigorous monitoring of adherence, lack of confirmation of actual intake, and insufficient reporting of adverse events, which was documented in only one trial. These limitations raise concerns about internal validity and safety assessment.

In addition, most trials were conducted in countries with mandatory vitamin E food fortification policies. This context likely reduced the prevalence of baseline deficiency and may have masked potential benefits of supplementation, as none of the included studies adequately characterized maternal vitamin E status at enrollment or stratified analyses according to deficiency. Consequently, the effects of supplementation in populations with confirmed deficiency or greater nutritional vulnerability remain unclear.

Regarding neonatal outcomes, although supplementation effectively increased maternal α‐TOH concentrations, no consistent improvements were observed in clinically relevant endpoints such as birth weight or gestational age. Isolated reports of increased neonatal mortality and congenital malformations among supplemented women were described in two studies, but the available evidence is insufficient to establish causality. These findings reinforce the need for cautious interpretation and underscore the uncertainty surrounding the clinical benefits of routine supplementation in unselected pregnant populations.

From a biological perspective, adequate maternal vitamin E status is essential for neonatal immune, pulmonary, vascular, and neurological development (Youness et al. 2022; da Silva et al. 2017; Traber and Manor 2012). Deficiency has been associated with impaired neonatal α‐TOH supply and adverse outcomes such as hemolytic anemia and bronchopulmonary dysplasia (Youness et al. 2022). Observational evidence also suggests links between low vitamin E levels and increased severity of preeclampsia, renal dysfunction, oxidative stress, and unfavorable neonatal outcomes (Duan et al. 2021; Hao 2024). Nevertheless, these associations do not necessarily translate into benefits from indiscriminate supplementation.

Importantly, vitamin E supplementation is not without potential risks. Evidence from large‐scale reviews indicates that high‐dose supplementation may exert pro‐oxidant effects, interfere with cellular signaling pathways, and increase bleeding risk due to the anticoagulant properties of α‐TOH (Rumbold et al. 2015). Consistent with this, previous meta‐analyses have shown no reduction in major pregnancy complications or maternal mortality (Kaye et al. 2025), further questioning the clinical utility of routine supplementation.

Taken together, these findings suggest that while vitamin E deficiency poses risks to maternal and neonatal health, indiscriminate supplementation, particularly in heterogeneous populations without confirmed deficiency, may offer limited benefit and potential harm. Preventive strategies should therefore prioritize individualized approaches, focusing on the identification of pregnant women with documented deficiency or those at higher socioeconomic and nutritional risk.

This review presents important strengths, including its focused assessment of maternal α‐TOH levels, exclusive inclusion of randomized controlled trials, comprehensive literature search without language restrictions, and application of standardized methodological and evidence‐grading frameworks. However, significant limitations must be acknowledged. The small number of available trials, substantial methodological and statistical heterogeneity, limited safety reporting, and the absence of recent studies or data from low‐ and middle‐income countries restrict the generalizability and robustness of the conclusions.

Overall, the current evidence should be considered exploratory and hypothesis‐generating. Future well‐designed, multicenter randomized controlled trials with larger sample sizes, standardized supplementation protocols, careful assessment of baseline nutritional status, and comprehensive safety monitoring are essential to clarify the true benefits and risks of vitamin E supplementation during pregnancy and to identify populations most likely to benefit.

## 5 Conclusions

This systematic review demonstrates that vitamin E supplementation during pregnancy consistently increases circulating maternal α‐tocopherol levels; however, it does not lead to clear or consistent clinical benefits in key neonatal outcomes, such as birth weight and gestational age. Despite the biological relevance of this increase, it does not translate into clinical improvements in general populations of pregnant women. Although the available evidence is limited and heterogeneous, these findings highlight the need for future well‐designed, adequately powered randomized controlled trials that include detailed assessment of baseline vitamin E nutritional status, standardized supplementation protocols, and systematic monitoring of adherence and safety in order to clarify the true role of vitamin E supplementation during pregnancy.

## Author Contributions

**Yvi Melo Sisinano:** methodology, investigation, data curation. **Adriana Augusto de Rezende:** supervision, writing – review and editing. **Karla Danielly da Silva Ribeiro:** design, supervision, review and editing. **Byanca Rodrigues Carneiro:** methodology, investigation, data curation, review and writing. **Anny Cristine de Araújo:** conception, design, methodology and review.

## Funding

This work was supported by Coordenação de Aperfeiçoamento de Pessoal de Nível Superior, Financial code 001.

## Ethics Statement

The authors have nothing to report.

## Conflicts of Interest

The authors declare no conflicts of interest.

## Supporting information


**Table S1:** PRISMA checklist.
**Table S2:** Search strategy used in systematic review.
**Table S3:** Excluded studies and the reasons for their exclusion.

## Data Availability

The data that support the findings of this study are available from the corresponding author upon reasonable request.
