# Supplementary material for: Efficacy of Vitamin E Supplementation During Pregnancy on the Vitamin E Nutritional Status of the Mother–Child Dyad: A Systematic Review and Meta‐Analysis of Randomized Controlled Trial
Source: Food Sci Nutr. 2026 Mar 22;14(3):e71677. doi: 10.1002/fsn3.71677 (PMC13093448; doi:10.1002/fsn3.71677)
Supplement: Supplementary file 1 — Table S1: PRISMA checklist. Table S2: Search strategy used in systematic review. Table S3: Excluded studies and the reasons for their exclusion. [file FSN3-14-e71677-s001.docx]

**SUPPLEMENTARY MATERIAL**

**Supplementary Table 1.**  PRISMA checklist.

| **Section and Topic** | **Item #** | **Checklist item** | **Location where item is reported (page)** |
| --- | --- | --- | --- |
| **TITLE** | | | |
| Title | 1 | Identify the report as a systematic review. | 1 |
| **ABSTRACT** | | | |
| Abstract | 2 | See the PRISMA 2020 for Abstracts checklist. | 1-2 |
| **INTRODUCTION** | | | |
| Rationale | 3 | Describe the rationale for the review in the context of existing knowledge. | 2-3 |
| Objectives | 4 | Provide an explicit statement of the objective(s) or question(s) the review addresses. | 3 |
| **METHODS** | | | |
| Eligibility criteria | 5 | Specify the inclusion and exclusion criteria for the review and how studies were grouped for the syntheses. | 4 |
| Information sources | 6 | Specify all databases, registers, websites, organisations, reference lists and other sources searched or consulted to identify studies. Specify the date when each source was last searched or consulted. | 4 |
| Search strategy | 7 | Present the full search strategies for all databases, registers and websites, including any filters and limits used. | Table S2 |
| Selection process | 8 | Specify the methods used to decide whether a study met the inclusion criteria of the review, including how many reviewers screened each record and each report retrieved, whether they worked independently, and if applicable, details of automation tools used in the process. | 4-5 |
| Data collection process | 9 | Specify the methods used to collect data from reports, including how many reviewers collected data from each report, whether they worked independently, any processes for obtaining or confirming data from study investigators, and if applicable, details of automation tools used in the process. | 5 |
| Data items | 10a | List and define all outcomes for which data were sought. Specify whether all results that were compatible with each outcome domain in each study were sought (e.g. for all measures, time points, analyses), and if not, the methods used to decide which results to collect. | 5 |
|  | 10b | List and define all other variables for which data were sought (e.g. participant and intervention characteristics, funding sources). Describe any assumptions made about any missing or unclear information. | 5 |
| Study risk of bias assessment | 11 | Specify the methods used to assess risk of bias in the included studies, including details of the tool(s) used, how many reviewers assessed each study and whether they worked independently, and if applicable, details of automation tools used in the process. | 6 |
| Effect measures | 12 | Specify for each outcome the effect measure(s) (e.g. risk ratio, mean difference) used in the synthesis or presentation of results. | 5  Tables 1 and 2 |
| Synthesis methods | 13a | Describe the processes used to decide which studies were eligible for each synthesis (e.g. tabulating the study intervention characteristics and comparing against the planned groups for each synthesis (item #5)). | N/A |
|  | 13b | Describe any methods required to prepare the data for presentation or synthesis, such as handling of missing summary statistics, or data conversions. | 5 |
|  | 13c | Describe any methods used to tabulate or visually display results of individual studies and syntheses. | 5 |
|  | 13d | Describe any methods used to synthesize results and provide a rationale for the choice(s). If meta-analysis was performed, describe the model(s), method(s) to identify the presence and extent of statistical heterogeneity, and software package(s) used. | 5 |
|  | 13e | Describe any methods used to explore possible causes of heterogeneity among study results (e.g. subgroup analysis, meta-regression). | N/A |
|  | 13f | Describe any sensitivity analyses conducted to assess robustness of the synthesized results. | N/A |
| Reporting bias assessment | 14 | Describe any methods used to assess risk of bias due to missing results in a synthesis (arising from reporting biases). | 6 |
| Certainty assessment | 15 | Describe any methods used to assess certainty (or confidence) in the body of evidence for an outcome. | 6 |
| **RESULTS** | | | |
| Study selection | 16a | Describe the results of the search and selection process, from the number of records identified in the search to the number of studies included in the review, ideally using a flow diagram. | 6  Figure 1 |
|  | 16b | Cite studies that might appear to meet the inclusion criteria, but which were excluded, and explain why they were excluded. | 6  Table S3 |
| Study characteristics | 17 | Cite each included study and present its characteristics. | 6-7 |
| Risk of bias in studies | 18 | Present assessments of risk of bias for each included study. | 8-9  Figure 3 |
| Results of individual studies | 19 | For all outcomes, present, for each study: (a) summary statistics for each group (where appropriate) and (b) an effect estimate and its precision (e.g. confidence/credible interval), ideally using structured tables or plots. | 8 Figure 2 |
| Results of syntheses | 20a | For each synthesis, briefly summarise the characteristics and risk of bias among contributing studies. | 9  Figure 3 |
|  | 20b | Present results of all statistical syntheses conducted. If meta-analysis was done, present for each the summary estimate and its precision (e.g. confidence/credible interval) and measures of statistical heterogeneity. If comparing groups, describe the direction of the effect. | N/A |
|  | 20c | Present results of all investigations of possible causes of heterogeneity among study results. | N/A |
|  | 20d | Present results of all sensitivity analyses conducted to assess the robustness of the synthesized results. | N/A |
| Reporting biases | 21 | Present assessments of risk of bias due to missing results (arising from reporting biases) for each synthesis assessed. | N/A |
| Certainty of evidence | 22 | Present assessments of certainty (or confidence) in the body of evidence for each outcome assessed. | 9  Table 3 |
| **DISCUSSION** | | | |
| Discussion | 23a | Provide a general interpretation of the results in the context of other evidence. | 9-10 |
|  | 23b | Discuss any limitations of the evidence included in the review. | 9-10 |
|  | 23c | Discuss any limitations of the review processes used. | 9-10 |
|  | 23d | Discuss implications of the results for practice, policy, and future research. | 11 |
| **OTHER INFORMATION** | | | |
| Registration and protocol | 24a | Provide registration information for the review, including register name and registration number, or state that the review was not registered. | 3-4 |
|  | 24b | Indicate where the review protocol can be accessed, or state that a protocol was not prepared. | 3-4 |
|  | 24c | Describe and explain any amendments to information provided at registration or in the protocol. | N/A |
| Support | 25 | Describe sources of financial or non-financial support for the review, and the role of the funders or sponsors in the review. | 12 |
| Competing interests | 26 | Declare any competing interests of review authors. | N/A |
| Availability of data, code and other materials | 27 | Report which of the following are publicly available and where they can be found: template data collection forms; data extracted from included studies; data used for all analyses; analytic code; any other materials used in the review. | 12 |

N/A: Not applicable. *From:* Page MJ, McKenzie JE, Bossuyt PM, Boutron I, Hoffmann TC, Mulrow CD, et al. The PRISMA 2020 statement: an updated guideline for reporting systematic reviews. BMJ 2021;372: n. 71. DOI: 10.1136/bmj.n71.

**Supplementary Table 2.** Search strategy used in systematic review.

| Database | Search equation |
| --- | --- |
| PubMED/MEDLINE | pregnant women OR women, pregnant OR pregnancy OR gestation OR pregnancies AND dietary supplement OR vitamin E OR tocopherols OR tocotrienols AND sham treatment OR placebo effect AND vitamin E deficiency OR alpha‐tocopherol OR beta-tocopherol OR gamma-tocopherol AND infant, newborn OR newborn infants OR newborns OR neonate OR neonates AND clinical trials OR clinical study |
| Web of Science | pregnant women OR women, pregnant OR pregnancy OR gestation OR pregnancies AND dietary supplement OR vitamin E OR tocopherols OR tocotrienols AND sham treatment OR placebo effect AND vitamin E deficiency OR alpha‐tocopherol OR beta-tocopherol OR gamma-tocopherol AND clinical trials |
| EMBASE | (“pregnant women” OR “women, pregnant” OR “pregnancy” OR “gestation” OR “pregnancies”) AND (“dietary supplement” OR “vitamin E” OR “tocopherols” OR “tocotrienols”) AND (“sham treatment” OR “placebo effect”) AND (“vitamin E deficiency” OR “alpha‐tocopherol” OR “beta-tocopherol” OR “gamma-tocopherol”) AND (“infant, newborn” OR “newborn infants” OR “newborns” OR “neonate” OR “neonates”) AND (“clinical trials” OR “clinical study”) |
| Scopus | “pregnant women” OR “women, pregnant” OR “pregnancy” OR “gestation” OR “pregnancies” AND “dietary supplement” OR “vitamin E” OR “tocopherols” OR “tocotrienols” AND “sham treatment” OR “placebo effect” AND “vitamin E deficiency” OR “alpha‐tocopherol” OR “beta-tocopherol” OR “gamma-tocopherol” AND “infant, newborn” OR “newborn infants” OR “newborns” OR “neonate” OR “neonates”AND “clinical trials” OR “clinical study” |
| LILACS/SciELO | ((Pregnant Women) OR (Women, Pregnant) OR (Pregnant) OR (Pregancy) OR (Gestation) OR (Pregnancies)) AND ((Dietary Supplement) OR (Vitamin E) OR (Tocopherols) OR (Tocotrienols)) AND ((Sham Treatment) OR (Placebo Effect)) AND ((Vitamin E Deficiency) OR (Alpha‐tocopherol) OR (Beta-tocopherol) OR (Gamma- tocopherol) OR (Infant, Newborn) OR (Newborn Infants) OR (Newborns) OR (Neonate) OR (Neonates)) AND ((Clinical Trials) OR (Clinical Study)) |
| Cochrane Library | (pregnant women) OR (women, pregnant) OR (pregnancy) OR (gestation) OR (pregnancies) AND (dietary supplement) OR (vitamin E) OR (tocopherols) OR (tocotrienols) AND (sham treatment) OR (placebo effect) d AND (vitamin E deficiency) OR (alpha‐tocopherol) OR (beta-tocopherol) OR (gamma-tocopherol) AND (infant, newborn) OR (newborn infants) OR (newborns) OR (neonate) OR (neonates) AND (clinical trials) OR (clinical study) |

**Supplementary Table 3.** Excluded studies and the reasons for their exclusion.

| Author (year) | Reference | Reason for exclusion |
| --- | --- | --- |
| Laskowska-Klita *et al.* (2004) | Laskowska-Klita T, Chełchowska M, Ambroszkiewicz J, et al. The effect of vitamin-mineral supplementation on vitamins D, A (beta-carotene) and E concentration in blood of matched maternal-cord pairs. *Przegl Lek.* 2004;61(7):755-9. PMID: 15792015. | Lack of open access and non-availability by authors upon request |
| Beazley *et al.* (2005) | Beazley D, Ahokas R, Livingston J, et al. Vitamin C and E supplementation in women at high risk for preeclampsia: a double-blind, placebo-controlled trial. *Am J Obstet Gynecol.* 2005;192(2):520-1. DOI: [10.1016/j.ajog.2004.09.005](https://doi.org/10.1016/j.ajog.2004.09.005) | Summary of a randomized clinical trial, published in the annals of scientific events |
| Rumbold *et al.* (2006) | Rumbold AR, Crowther CA, Haslam RR. Et al. Vitamins C and E and the risks of preeclampsia and perinatal complications. *New England Journal of Medicine* 2006;354(17):1796-1806. DOI: 10.1056/NEJMoa054186 | Lack of data to perform the analysis, absence of alpha-tocopherol values |
| Spinnato (2006) | Spinnato JA. New therapies in the prevention of preeclampsia. *Current Opinion in Obstetrics and Gynecology*. 2006;18(6):601-604. DOI: [10.1097/01.gco.0000247393.86968.e6](https://doi.org/10.1097/01.gco.0000247393.86968.e6) | Wrong study design, review study |
| Rumbold, Crowther, Haslam (2008) | Rumbold A, Duley L, Crowther CA, Haslam RR. Antioxidants for preventing pre-eclampsia. *Cochrane Database Syst Rev.* 2008(1):CD004227. DOI: [10.1002/14651858.CD004227.pub3](https://doi.org/10.1002/14651858.cd004227.pub3) | Wrong study design, review study |
| Rahimi *et al.* (2009) | Rahimi R, Nikfar S, Rezaie A, et al. A meta-analysis on the efficacy and safety of combined vitamin C and E supplementation in preeclamptic women. *Hypertens Pregnancy.* 2009;28(4):417-34. DOI: [10.3109/10641950802629667](https://doi.org/10.3109/10641950802629667) | Wrong study design, meta-analysis |
| Greenough *et al.* (2010) | Greenough A., Shaheen SO, Shennan A, et al. Respiratory outcomes in early childhood following antenatal vitamin C and E supplementation. *Thorax*. 2010;65(11):998-1003. DOI: [10.1136/thx.2010.139915](https://doi.org/10.1136/thx.2010.139915) | Lack of data to perform the analysis, absence of alpha-tocopherol  values |
| Gungorduk *et al.* (2013) | Gungorduk K, Asicioglu O, Gungorduk OC, et al. Does vitamin C and vitamin E supplementation prolong the latency period before delivery following the preterm premature rupture of membranes? A randomized controlled study. *Am J Perinatol.* 2014;31(3):195-202. DOI: [10.1055/s-0033-1343774](https://doi.org/10.1055/s-0033-1343774) | Lack of data to perform the analysis, absence of alpha-tocopherol values |
| Rumbold, Crowther (2005) | Rumbold A, Crowther CA. Vitamin E supplementation in pregnancy. *Cochrane Database Syst Rev.* 2005;(9). DOI: [10.1002/14651858.CD004069.pub2](https://doi.org/10.1002/14651858.cd004069.pub2) | Wrong study design, review study |
| Taghizadeh *et al.* (2016) | Taghizadeh M, Jamilian M, Mazloomi M, et al. A randomized-controlled clinical trial investigating the effect of omega-3 fatty acids and vitamin E co-supplementation on markers of insulin metabolism and lipid profiles in gestational diabetes. *J Clin Lipidol.* 2016;10(2):386-93. DOI: [10.1002/14651858.CD004069.pub2](https://doi.org/10.1002/14651858.cd004069.pub2) | Wrong study design, review study |
| Jamilian *et al.* (2017) | Jamilian M., Dizaji SH, Bahmani F, et al. A randomized controlled clinical trial investigating the effects of omega-3 fatty acids and vitamin E co-supplementation on biomarkers of oxidative stress, inflammation and pregnancy outcomes in gestational diabetes. *Canadian journal of diabetes*. 2017;41(2): 143-149. DOI: [10.1016/j.jcjd.2016.09.004](https://doi.org/10.1016/j.jcjd.2016.09.004) | Lack of data to perform the analysis, absence of alpha-tocopherol values |
| Haider, Bhutta (2017) | Haider BA, Bhutta ZA. Multiple‐micronutrient supplementation for women during pregnancy. *Cochrane Database Syst Rev.* 2017;(4). DOI: [10.1002/14651858.CD004905.pub5](https://doi.org/10.1002/14651858.cd004905.pub5) | Wrong study design, review study |
| Ostadmohammadi *et al.* (2019) | Ostadmohammadi V, Samimi M, Mobini M, et al. O efeito da cossuplementação de zinco e vitamina E no estado metabólico e sua expressão gênica relacionada em pacientes com diabetes gestacional. *The Journal of Maternal-Fetal & Neonatal Medicine*. 2019;32(24):4120-4127. DOI: [10.1080/14767058.2018.1481952](https://doi.org/10.1080/14767058.2018.1481952) | Lack of data to perform the analysis, absence of alpha-tocopherol values |
